# Thermal Imaging of the Tongue Surface as a Predictive Method in the Diagnosis of Type 2 Diabetes Mellitus

**DOI:** 10.3390/s24082447

**Published:** 2024-04-11

**Authors:** Daria Wziątek-Kuczmik, Antoni Świątkowski, Armand Cholewka, Aleksandra Mrowiec, Iwona Niedzielska, Agata Stanek

**Affiliations:** 1 Department of Cranio-Maxillofacial Surgery, Medical University of Silesia, 40-055 Katowice, Poland; dariakuczmik@interia.pl (D.W.-K.); antekswiatkowski99@gmail.com (A.Ś.); niedzielska.konsultant@wp.pl (I.N.); 2 Faculty of Science and Technology, University of Silesia, 40-007 Katowice, Poland; 3 Department and Clinic of Internal Medicine, Angiology and Physical Medicine, Faculty of Medical Sciences in Zabrze, Medical University of Silesia, 41-902 Bytom, Poland

**Keywords:** tongue, type 2 diabetes mellitus, dynamic infrared thermal image, tongue temperature, imaging method, glycated hemoglobin level

## Abstract

Over the past 20 years, the high prevalence of diabetes has become a global public health problem. Background: The objective of this study was to develop a non-invasive screening method for diabetes which will enable the detection of the disease at an early stage. Methods: This study included 63 adult patients of both sexes: 30 patients with type 2 diabetes (t2DM) and 33 healthy volunteers. The temperature distribution on the tongue’s dorsum and apex surface was studied in patients after a mouth-cooling procedure had been introduced. The study used an FLIR T540 thermal imaging camera. An analysis of the correlation between the ∆T values of the tongue dorsum and apex and the glycated hemoglobin (HbA1c) level was performed. Results: The median of the average dorsum temperature measured 10 min after mouth rinsing was almost 0.8 [°C] lower than for healthy individuals. Also, studies showed a positive average correlation with a Pearson coefficient of r = 0.46 between the HbA1c level and the ∆T of the tongue dorsum. Conclusions: Tongue temperature measured using the IRT showed a correlation with standard biochemical parameters; it may also differentiate patients and constitute a specific screening method for patients with t2DM.

## 1. Introduction

Diabetes is a chronic metabolic disease characterized by elevated levels of blood glucose (or blood sugar) which, over time, lead to serious damage to the heart, blood vessels, eyes, kidneys, and nerves. The most common variety is type 2 diabetes (t2DM), usually found in adults, which occurs when the body becomes resistant to insulin or does not make enough insulin. In the past three decades, the prevalence of t2DM has increased dramatically in countries of all income levels. Type 1 diabetes, once known as juvenile diabetes or insulin-dependent diabetes, is a chronic condition in which the pancreas produces little or no insulin by itself. For people living with diabetes, access to affordable treatment, including insulin, is critical for their survival. There is a globally agreed-upon target to halt the rise in diabetes and obesity by 2025 [1].

The WHO’s global plan for 2013–2030 includes strengthening the prevention and control of diabetes. It plans to stop the increase in the incidence of diabetes and obesity and provide 50% of eligible individuals treatment and 80% availability of affordable technologies and basic medicines. Noting that the growing number of people with diabetes is strongly linked to the inadequate prevention of risk factors underlying diabetes, such as overweight and obesity, an unhealthy diet, physical inactivity, and smoking, and is also linked to socio-economic status and social impacts [1].

It is estimated that over 420 million people worldwide currently suffer from diabetes, and this number will increase to 578 million by 2030 and 700 million by 2045. Ensuring high-quality primary health care; safe, effective, affordable, and essential medicines; diagnostics; and technology in universal health care is a priority [1].

The WHO Global Diabetes Compact’s initiative at the 2021 World Diabetes Summit was to reduce the risk of diabetes by ensuring that all people diagnosed with diabetes have access to equitable, comprehensive, affordable, and high-quality treatment, guaranteeing safe, effective diagnostics and medical technology by qualified health care professionals [2].

Long-term hyperglycemia can cause multisystem damage and results in retinopathy, neuropathy, nephropathy, macrovascular disease, and delayed wound healing. In addition to these complications, diabetes mellitus also has damaging effects on the oral cavity. It leads to changes in the periodontal tissues and oral mucosa, changes the function of the salivary glands, and increases the risk of caries [3,4,5].

One of the complementary diagnostic tools available that has proven effective in the control of diabetes, and especially the diabetic foot, is infrared thermography (IRT) [6,7,8,9,10].

IRT is a safe, repeatable, contactless, and non-invasive procedure that measures and maps the temperature distribution radiating from body surfaces. An infrared camera identifies and monitors the amount of radiation emitted and translates this value into a temperature value. These projections allow for the identification of heat radiating away from the body and produce images with specific physiological thermal patterns that can be collected according to specific standards. Based on the results obtained, appropriate information about the patient’s health condition can be provided, which can accelerate and help patients make treatment decisions [6,7].

The assessment of tongue temperature using IRT has been reported to be used in the diagnosis of diabetes, anemia, and coronary artery disease based on local changes in temperature secondary to changes in blood flow [11,12,13,14]. This work is a continuation of the author’s method of obtaining thermal imaging measurements of the temperature distribution on the surface of the tongue, which can be used in the early diagnosis of general diseases, including diabetes, which manifest in fluctuations in internal temperature [15].

## 2. Materials and Methods

### 2.1. Patients with Diabetes and Healthy Subjects

This clinical study included 63 adult patients of both sexes, 30 of whom were diagnosed with type 2 diabetes mellitus and were hospitalized at the Department and Clinic of Internal Medicine, Angiology and Physical Medicine of Specialist Hospital No. 2 in Bytom. The control group consisted of 33 healthy volunteers without additional diseases who were patients at the Maxillofacial Surgery Department of the Medical University of Silesia in Katowice.

Patients were eligible for this study based on medical records and a panel of laboratory tests. All patients underwent a clinical examination that included a medical history and an oral examination by a dentist. A self-administered medical history questionnaire was developed for the study, including age, sex, time since the diagnosis of diabetes, medications, stimulants, allergies, and comorbidities. The detailed medical history was supplemented with a question about symptoms resulting from the underlying disease, such as tingling sensations in the feet and hands, a lack of healing wounds in the limbs, and symptoms of limb ischemia.

The dental history obtained included information on the frequency of visits and the last visit to the dentist and questions about gingival bleeding, the impaired healing of mucosal wounds, dry mouth, and the possible use of dentures.

A detailed examination of the oral cavity was carried out: the condition of the oral mucosa and the periodontium and color, moisture, and pathological changes was assessed. The tongue was carefully examined, and the dorsal surface of the tongue was assessed for hypertrophy or atrophy of the lingual papillae and for eruptions and plaque.

The dental chart included information about caries and non-carious decay, retained roots, missing teeth, oral hygiene, and dental plaque.

The inclusion criteria were adult patients of both sexes with a t2MD diagnosis and an HbA1c level measured in erythrocytes. All patients consumed a diet with a low glycemic index which was rich in protein, vegetables, and fish.

Patients were excluded if they had an elevated body temperature, an active inflammatory process of a bacterial or viral origin, or a neoplastic process in the head and neck, as well as any comorbidities such as hypertension, anemia, cardiovascular disease, hypo- and hyperthyroidism, or dyslipidemia.

The tongue is a strongly muscled movable organ, resting freely on the floor of the mouth, and is easy to perform a non-contact examination of the tongue. In our work, we focused on the assessment of tongue temperature because its value significantly correlates with the blood perfusion rate and metabolic processes. To describe anomalies, the tongue was divided into two sectors: the dorsum (TD) and the apex (AT).

This study’s protocol abided by the principles of the Declaration of Helsinki and was approved by the Ethics Committee of the University of Medical University of Silesia under the reference PCN/CBN/0052/KB1/67/II/22/23. The patients were all volunteers and provided their informed consent prior to their inclusion in the study.

### 2.2. Infrared Thermal Imaging Measurements

The temperature of the tongue in the studies was determined according to international standards of thermal imaging in medicine [8]. A period of thermal equilibrium which is required before thermal imaging measurements could be also taken to achieve a level of stability in blood pressure and body temperature. This time was 20 min of waiting at rest. The patients were always examined by the same team of investigators at a similar time of day and season in a room with a constant temperature of 21 + 0.5 °C and humidity between 50% and 60%. The thermal camera was calibrated by a black body with possibility of setting its temperature within the range occurring in the human organism. So, the detector’s accuracy was checked in the study room’s environmental conditions. The human skin surface exhibits exceptional radiative properties, boasting an emissivity of 0.98. In comparison, a perfect black body radiator, which possesses maximal absorption and radiation capabilities, is standardized to have uniform emissivity [16]. The exposure room had no air conditioning and closed windows and doors to eliminate airflow around the subject. Body temperature, blood pressure, heart rate, and BMI were measured.

The examination was carried out in a diagnostic chair, with the patient’s head resting on the headrest and the chin on a plane parallel to the floor. The study of the tongue was conducted using a thermal imaging camera, an FLIR T 540 (Teledyne FLIR LLC, Wilsonwille, OR, USA), with a sensitivity of <0.03 K. The Flir T540 camera has a true maximal sensor resolution of 464 × 348 pixels (161,472 measurement points). Its thermal sensitivity/noise equivalent thermal detection (NETD) is as high as <40 mK at 30 °C. This type of detector is an uncooled microbolometer. The spatial resolution is 0.90 mrad/pixel at 24°, and the spectral range is 7.5–14 µm. It gives the possibility of measuring components down to a 71 µm/pixel spot size. The accuracy of a modern thermal imaging camera is ±2 °C or ±2%, which was considered during the image analysis. This is a normal measurement characteristic of a 2D thermal detector. However, such a value refers to the temperature reading of the detector, which heats up during operation. Therefore, the camera was always switched on, and an inhomogeneity correction was performed before each measurement to ensure that the measurement was taken at the same point in the detector operating curve. After this step, the reading temperature should be almost strictly dependent on the tongue temperature.

The camera was positioned perpendicular to the skin surface and 0.4 ± 0.05 m away from the body in accordance with established standards for infrared medical diagnosis [11].

For cold provocation, the patient was instructed to rinse their mouth with water at 22 °C for 60 s. Moreover, during the measurements, the patient was asked to breathe through their nose so as not to disturb the measurement by breathing through their mouth.

This was followed by closing the mouth for 10 min before the measurement, leaving the tongue stationary and with silence. Two test cycles were performed at 2 and 10 min each with two full measurement cycles, in line with previous in-house studies, according to standards introduced in the literature [11,12].

Thermal imaging of the first test (I) was performed after 2 min, with the mouth wide open, having the tongue lie freely on the floor of the mouth. Similarly, a second test (II) was performed after a further 8 min—the muscle was relaxed, with the tip of the tongue pointing slightly downward. During both tests, the patient had their mouth open for 5 s.

### 2.3. Other Examined Parameters

Blood pressure and body temperature were measured by the same researchers, always at similar early afternoon hours, according to an established algorithm.

For at least 30 min before the measurements, the patient did not drink coffee or perform intense physical exercise. The measurement was taken no earlier than approximately 60 min after eating a large meal. For 3–5 min before the measurement, the patient remained in a sitting position in a quiet environment. The patient’s feet were on the ground, and the back and upper limbs were relaxed when the blood pressure was measured. During the measurement, the upper limb should be relaxed and the palm of the hand facing upwards. After selecting the appropriate size of blood pressure monitor cuff, measurements were taken with an electronic blood pressure monitor. Body temperature was always measured with a non-contact thermometer on the forehead from a distance of 2–3 cm with the patient at rest and in a relaxed body position. The body mass index (BMI) was determined by reading the patient’s height and weight from medical documentation and using a standard equation. The method for measuring glycated hemoglobin (HbA1c) was based on the immunoturbidimetric method. Patients with tDM2 were treated with oral hypoglycemic drugs. Most often, it was therapy with biguanide derivatives (metformin).

### 2.4. Statistical Analysis

In order to compare differences between the initial values of the temperature parameters obtained due to the mouth-rinsing procedure in both groups of subjects involved in this study, an independent (unpaired)-samples Student’s t-test or Mann–Whitney U test was used, depending on the homogeneity of variances and the normality of data distribution. The correlation was performed by using the Pearson coefficient. Significant statistical differences were considered when *p* < 0.05.

## 3. Results

### 3.1. Characteristics of Patients with t2DM and Healthy Subjects

The gender distribution was as follows: 33 males (52.3%) and 30 females (47.7%). The age of the subjects ranged from 22 to 88 years. The mean age was 51.2 years. The distribution of BMI values was as follows 32.3% were of normal weight, and 43.1% were overweight. First-degree obesity, second-degree obesity, and extreme obesity were as follows: 18.5%, 0%, and 4.6%. The mean BMI and blood pressure were 27.29 and 123.8/74.4, respectively (Table 1 and Table 2).

The research group comprised 30 Caucasian patients aged 35–88; the median age was 70. Most of these subjects were of retirement age. Patients under 65 years of age in the case of men and under 60 years of age in the case of women mainly belonged to the middle class of society (Table 1 and Table 2).

In the study group, the average time from diagnosis was 13.05 years. One of the patients had been treated for t2DM for 50 years, while two patients had had a diagnosis of the disease for less than a year. The characteristics of the research group are presented in Table 1 and Table 2. The characteristics of the patients with t.2DM and the healthy volunteers are presented in Table 1. The patients with t2DM were dived into two subgroups according to the criterion of diabetes control (HbA1c ≤ 7% compensated; HbA1c > 7% decompensated) (Table 2) [17].

### 3.2. Thermographic Diagnosis of Patients with Type 2 Diabetes

**Figure 1:**
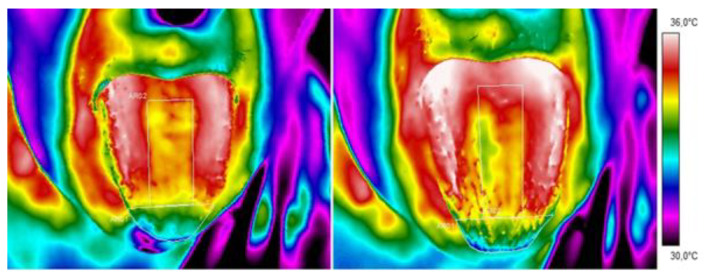
Thermal images of a representative patient (from the diabetes group) obtained 2 and 10 min after mouth rinsing.

**Figure 2:**
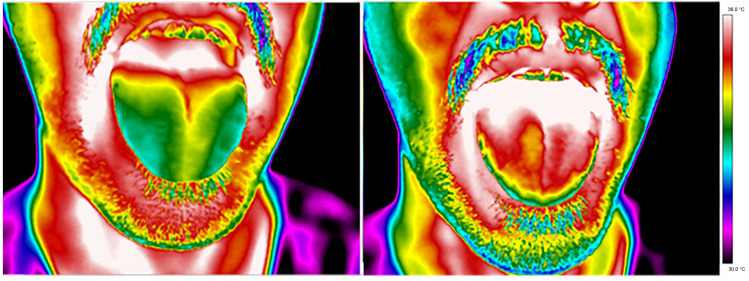
Thermal images of a representative healthy volunteer (from the control group) obtained 2 and 10 min after mouth rinsing.

Thermal images of a representative patient’s tongue were obtained from the diabetes group and control group and are presented in Figure 1 and Figure 2, respectively.

A qualitative analysis of the presented thermal images indicates that the tongue temperature differs due to mouth rinsing, which is obvious because the water temperature cooled the mouth. However, 10 min after mouth washing, the difference between the thermal behaviour of diabetic and healthy tissue can be seen. The images presented show faster dynamics of an increase in tongue temperature for healthy volunteers and not for patients. For better insight into the problem, a statistical analysis was performed. In the first stage, the mean temperatures of the dorsum and tip of the tongue were compared between a group of patients with t2DM and a control group (study 1).

**Figure 3:**
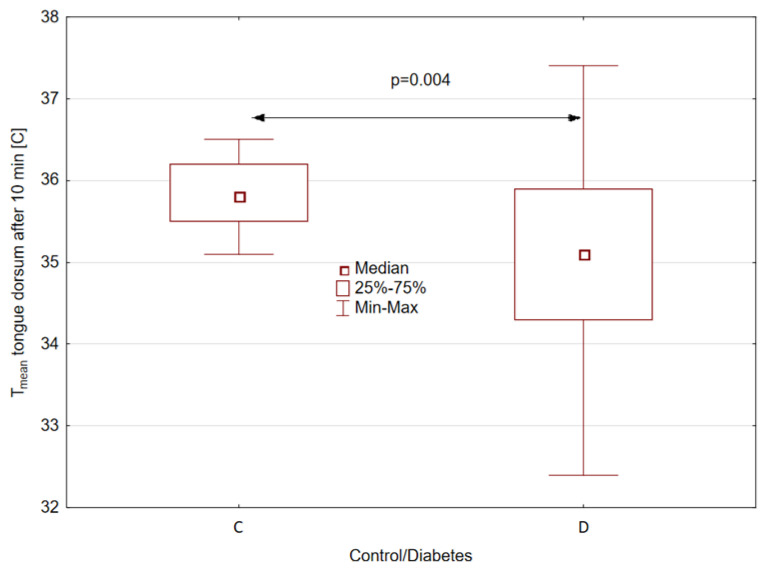
Mean temperature difference in tongue dorsum between control (C) and study groups (D—diabetes), obtained 10 min after rinsing the mouth with water.

Statistically significant (*p* = 0.004) differences were obtained between the mean temperatures of the tongue dorsum of patients and healthy volunteers for the temperature measured 10 min after mouth rinsing and for the temperature difference between 2 and 10 min after mouth rinsing whereby for patients with t2DM, the median of the average temperature of the tongue dorsum measured 10 min after mouth rinsing is almost 0.8 [°C] lower, as shown in Figure 3.

**Figure 4:**
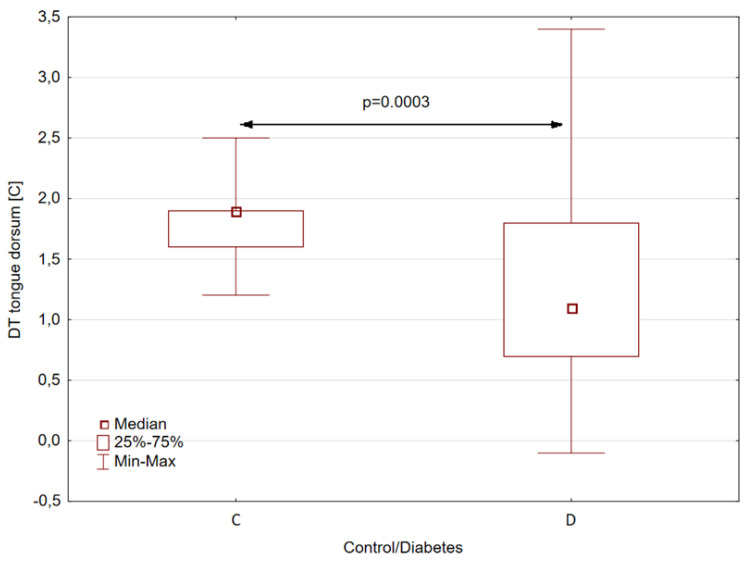
Temperature difference of tongue dorsum for control (C) and study groups (D—diabetes) obtained between 2 and 10 min after rinsing the mouth with water.

Similarly, statistically significant differences between patients and healthy subjects were obtained for the parameter DT, defined as the difference between the average temperatures of the back of the tongue 2 and 10 min after rinsing the mouth with water. In this case, for patients with type II diabetes, the median DT is almost 0.8 [°C] lower than for healthy volunteers, as shown in Figure 4. The statistical significance coefficient for this difference was as high as 0.0003.

Another aim of the performed study was to check if there was any dependence between the HbA1c level in erythrocytes and temperature in the group of patients with diabetes. That was why in the case of the patient group, the HbA1c level in erythrocytes was also marked (study 2).

The results obtained were collected and correlated with the values of the temperature parameters: the mean temperatures of the dorsum and apex of the tongue 2 and 10 min after rinsing the mouth, and temperature difference parameters between 2 and 10 min after rinsing the mouth. The correlation results obtained are summarized in Table 3.

Correlations between HbA1c levels and the temperature parameters of the tongue were performed for 27 cases of patients with t2DM.

Statistically insignificant weak, positive correlations were obtained for the mean temperature of the dorsum of the tongue 10 min after rinsing the mouth, for the tongue apex after 2 and 10 min, and for the DT parameter for the tongue apex, which could at most suggest some dependence that would need to be tested using a much larger study group.

**Figure 5:**
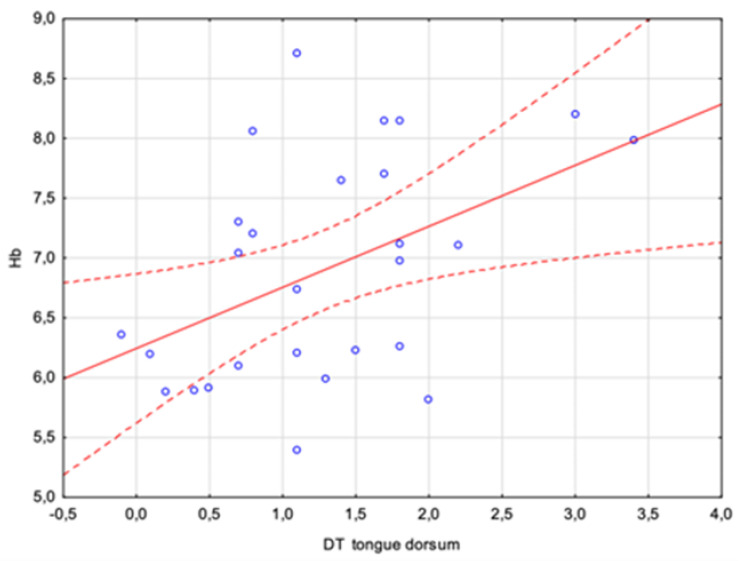
Correlations (r = 0.46) between the glycated hemoglobin (HbA1c) concentration and the difference in the tongue dorsum temperature between 2 and 10 min after rinsing the mouth.

**Figure 6:**
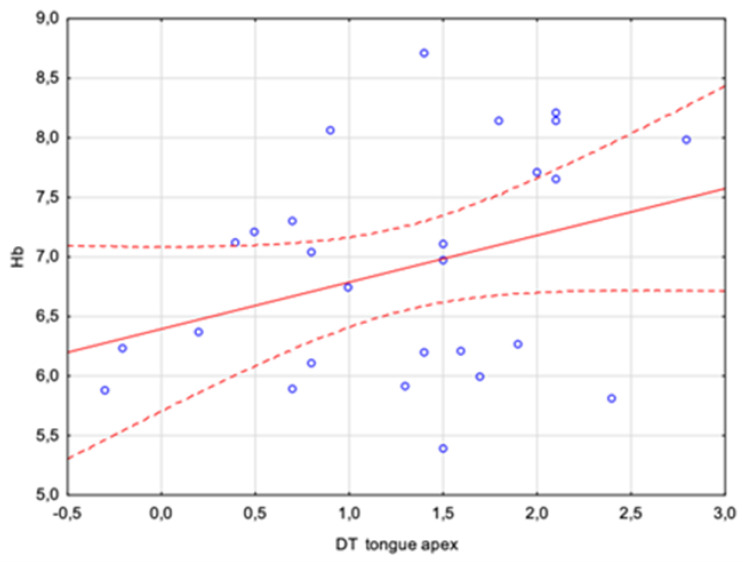
Correlations between the glycated hemoglobin (HbA1c) concentration and the difference in tongue apex temperature between 2 and 10 min after rinsing the mouth.

However, attention should be paid to a parameter that already differentiates between sick and healthy individuals as an independent measure, i.e., the tongue dorsum DT. The analyses carried out showed a positive average correlation with a Pearson coefficient of r = 0.46. There was a statistically significant relation between the HbA1c concentration and the difference in the tongue dorsum temperature between 2 and 10 min after rinsing the mouth. This is a result that may quite clearly indicate the possibility of assessing certain blood parameters using a properly performed tongue temperature measurement. The relationship is shown in Figure 5 and Figure 6.

In addition, preliminary correlations suggest the use of thermal imaging as a method of diagnostic procedure, which includes pre-cooling the tongue by rinsing the mouth, in the evaluation of other parameters hitherto calculated only from blood. Of course, these are preliminary measurements, and the study group is small in size, but the results seem to be unambiguous, at least at this stage of the research. However, an additional analysis between the dorsum temperature and HbA1c level is required.

**Figure 7:**
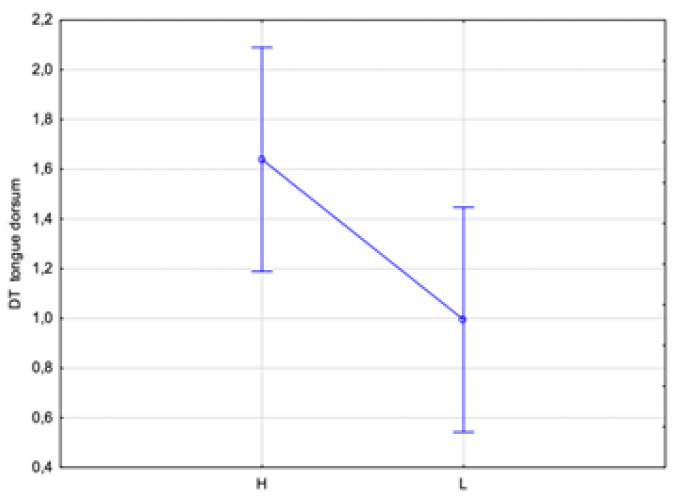
Temperature difference of tongue dorsum for patient group, characterized and divided according to HbA1c level, assuming L < 7% and H ≥ 7%, obtained between 2 and 10 min after rinsing mouth with water.

**Figure 8:**
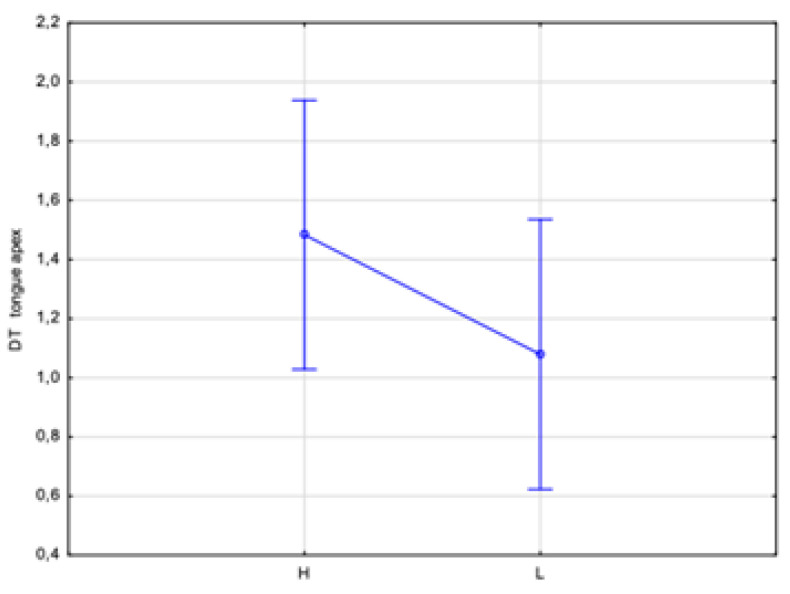
Temperature difference of tongue apex for patient group, characterized and divided according to HbA1c level, assuming L < 7% and H ≥ 7%, obtained between 2 and 10 min after rinsing mouth with water.

An additional analysis of the correlation between the DTs of the dorsum and apex tongue temperatures with the HbA1c level was performed. Two ranges of the HbA1c level were assumed: low, indicated by L < 7%, and high, indicated by H ≥ 7%. This division showed statistically significant differences in temperature (*p* < 0.05) between the assumed low and high HbA1c levels obtained for both the dorsum and apex DTs by almost 0.6 °C and 0.4 °C, respectively (Figure 7 and Figure 8). Thus, such a test should unambiguously indicate whether a patient has glycated HbA1c equalized.

Such a test, in contrast to the correlations presented above, may be decidedly more useful as a screening test for a patient admitted to the ward. This test will indicate that a patient has a high level of HbA1c without attempting to assess the percentage level, as the authors attempted to show for the correlations in the first two graphs.

## 4. Discussion

The fact that one in two adults with t2DM remains undiagnosed and four in five adults with diabetes live in low- and middle-income countries indicates the global scope of the problem. This paper is a follow-up to a report published in the *Journal of Clinical Medicine* in 2023 on using infrared thermography to measure tongue temperature for the initial diagnosis of general diseases [15]. However, in this study, the authors measured tongue temperature in patients with t2DM.

The great advantage of thermal imaging is that it is both non-contact and non-invasive. With a remote temperature sensor, the camera only captures the natural thermal energy emitted by the body. Since no harmful energy is used in the imaging process, it is suitable for repeated examinations. Infrared thermography (IRT) is a technique that enables the easy visualization of temperature at a specific location and records it continuously in the form of high-resolution images. The Flir T540, utilized for these tests, features a genuine sensor resolution of up to 464 × 348 pixels (equating to 161,472 measuring points). It offers a measurement accuracy of ±2 °C or ±2% of the reading.

IRT is capable of mapping the temperature distribution in areas of interest, providing a profile that can be obtained almost instantly, regardless of location and time [4,5,6].

In order to obtain accurate and reliable infrared thermal images, an established clinical testing methodology with several basic conditions is indispensably required [15,18].

To conduct imaging, it is necessary for a patient to achieve thermal equilibrium in an examination room with a constant ambient temperature, humidity, air circulation, and lighting. Twenty minutes are generally sufficient for a relaxed and acclimatized patient to reach a reasonable level of blood pressure and skin temperature stability [5]. Therefore, our qualified medical personnel strictly adhered to the thermographic exposure algorithm.

Abnormalities in the thermographic patterns of the hands and feet have been observed in patients with diabetes due to reduced blood circulation, which leads to a decrease in body temperature [3,4,5,6,7,8]. To visualize the metabolic state more accurately, attempts have been made to cold-challenge the test area. The method introduced by Jiang et al. in 2002 has been found to be highly specific [5,19,20].

The tongue, along with the hands and feet, is now being used as a diagnostic organ and test subject for IRT measurements. Thirunavukkarasu, Xie, and Nicolas-Rodrigues have used thermography for the prognosis of general diseases like diabetes mellitus, anemia, and circulatory disorders. It has also been beneficial in evaluating conditions affecting the oral cavity, such as lichen planus and burning mouth syndrome [11,12,13,21,22].

Considering that the tongue is well vascularized and that its surface temperature reflects the internal temperature carried by blood flow, this organ is of significant clinical importance. Tongue temperature measured by IRT shows a correlation with patient age and sex, biochemical parameters, differential symptoms, and signs of disease [11,12,13,14,15,21,23].

T2DM has a negative impact on vascular biology due to hyperglycemia and oxidative stress. Endothelial dysfunction is believed to be the first step in the adverse sequence of events leading to the atherosclerotic process. The term “endothelial dysfunction” refers to a condition in which the endothelium loses its physiological properties, i.e., the ability to dilate vessels, regulate fibrinolysis, and counteract aggregation. When endothelial integrity is lost, vasoconstriction occurs instead of vasodilation. Hyperglycemia induces endothelial cell apoptosis, exposing the inner membrane and leading to its dysfunction, arterial stiffness, and diabetic complications. Consequently, patients with diabetes have a two-to-four times higher risk of coronary artery disease and cerebrovascular disease compared to those without diabetes. Therefore, it is essential to closely monitor these patients for vascular complications and arterial stiffness caused by poor blood circulation [24,25,26].

With time, the risk of microvascular disease also increases, indicating the critical role of hyperglycemia in the etiology of small vessel disease leading to neuropathy, nephropathy, or retinopathy. Therefore, even low but supraphysiological blood glucose concentrations are directly responsible for intracellular disorders [25]. Our study proves that impaired tongue microcirculation leads to hypoxia and impaired thermoregulation, as manifested by temperature fluctuations.

According to the 2023 recommendations of the Polish Diabetes Association, diabetes diagnosis and screening are currently based on an invasive biochemical method involving routine, invasive blood sampling. It is recommended to determine fasting blood glucose and perform an oral glucose tolerance test; additionally, the value of the HbA1c level can be used. These tests should be performed once every three years over the age of 45 and annually in people at risk [17].

It is therefore crucial to find a way to screen for diabetes that is non-invasive and can detect the disease early on in a larger population.

The authors believe that infrared thermography is a promising method for predicting and monitoring the progression of t2DM. We provoked the tongue musculature of the participants with room-temperature water, which led to a significant thermoregulatory disturbance between the control and study groups (study 1). The median of the mean temperature of the tongue dorsum was 0.8 C lower after 10 min of rinsing the mouth for patients with t2DM. Similarly, the difference between the mean temperature of the back of the tongue at 10 and 2 min after mouth washing is 0.9 C lower for people with diabetes than healthy subjects. This test differentiates patients with diabetes from control volunteers and is very promising due to its simplicity and the short time required to perform the procedure.

Given that the subjects with diabetes were inpatients and underwent a panel of biochemical tests, including an assessment of HbA1c, we evaluated the correlation between its concentration and the temperature parameters of the tongue (study 2). The temperature of the tongue dorsum was found to differentiate patients suffering from diabetes from healthy volunteers. A statistically significant correlation was observed between the glycated hemoglobin concentration and the difference in tongue dorsum temperature at 10 and 2 min after mouth washing.

According to our research, the dorsum, or the back of the tongue, is the most representative area. Jiang et al. [13] found that tongue temperature was unevenly distributed, with the highest temperature at the root of the tongue and gradually decreasing towards the center. They also observed that the tongue tip had the lowest temperature [13].

Baek et al. [23] also found that temperatures on both sides of the tongue root were higher compared to other areas. This could be due to the vasculature of the lingual artery, which originates from the external carotid artery and runs along both sides of the tongue. Therefore, they suggest that the evaluation of tongue temperature should be based on the course of the lingual artery.

The hemoglobin A1c test is used to evaluate a person’s level of glucose control. The test shows an average of the blood sugar level over the past 90 days and presents a percentage. The test can also be used to diagnose diabetes [27].

An integral part of this study was the analysis of the correlation between the DTs of the dorsum and apex tongue temperatures with the HbA1c level. Two ranges of the HbA1c level were assumed: low, set at <7%, and high, set at H ≥ 7%. It seems that this could be easy to use in a practice screening test due to the obtained statistically significant differences in temperature between the assumed low and high glycated hemoglobin levels of the dorsum and apex DTs by almost 0.6 °C and 0.4 °C, respectively. The assessment at an HbA1c level of 7% was not coincidental but in accordance with the current guidelines of the Polish Diabetes Association, which define the target value for HBA1c as 7%. The results obtained indicate that thermal imaging with tongue cooling could be used to estimate other biochemical parameters hitherto determined only from blood samples. Non-linear regression models or machine learning algorithms were not used during this analysis due to the limited number of participating patients. It is planned to perform this type of analysis in future studies.

Of course, these are preliminary observations, and the study group is small. However, the results seem to be conclusive, at least at this stage of research.

There has been a worrying 5% rise in premature mortality due to diabetes in the last 20 years [1,2]. We hope that IRT, a simple and non-invasive diagnostic method, can be a good option for screening large populations.

## 5. Integrating Thermal Imaging of the Tongue into Public Health Screening Programs for T2DM

Type 2 diabetes is a growing epidemic which poses a significant public health burden due to increased morbidity and mortality. The primary goal of this study was to develop a method that would be applicable to screening tests in dental offices. Coordinated efforts by physicians and dentists are a cost-effective way to improve metabolic control. Bearing in mind the relationship between oral and other non-communicable diseases, the strongest association was observed with systemic diseases, especially T2DM. In addition to pharmacological treatment, the World Health Organization recommends a healthy diet, physical activity, and regular screening, including dental examinations, to eliminate the inflammatory factor to improve glycemic control. Therefore, the introduction of a low-cost, non-invasive method of assessing tongue temperature using IRT for glycemic control appears to be an available, effective, and low-cost method in countries at all levels of education, especially in low- and middle-income and the poorest areas.

This method uses data obtained from thermal images and its calculated temperature parameters correlate with standard biochemical parameters, including HgA1c, so it can differentiate patients and constitute a specific screening method for patients with t2DM. Such diagnostics can also be performed by a health care professional who is not a doctor, e.g., a certified diabetes specialist whose availability and labor costs are significantly competitive compared to medical services. It should be noted that modern thermal cameras are becoming less expensive, so they are available for primary care medicine physicians. Moreover, when machine learning is involved and the whole process is automatic, the screening method will be very user-friendly. Further clinical studies are necessary.

## 6. Conclusions

This research was conducted using the authors’ test technique of exposing the tongue to cold water in patients diagnosed with type 2 diabetes, which can significantly aid in population screening. The findings suggest that the temperature of the tongue can distinguish patients and offer a specific screening test for type 2 diabetes.

The tongue temperature measured by IRT significantly correlates with standard biochemical parameters. It provides information on the level of glycated hemoglobin recommended for monitoring glycemic control and thus for assessing the risk of diabetes complications.

Further research is needed to determine the potential of the IRT test for the prediction of morbidity and the control of type 2 diabetes. Research on thermal imaging of the tongue among patients with type 2 diabetes may lead to the development of a consistent screening method for the disease in the future. The test is non-invasive, does not involve ionizing radiation, and is mobile, cost-effective, and easy to use, which makes it accessible to every patient.

## Figures and Tables

**Table 1:** Parameters of the research (patients with t2DM) and control groups.

Parameters	Research Group (n = 30)	Control Group (n = 33)
Male/female ratio	17/13	16/17
Age (years), mean (±SD)	69.12 (±8.31)	33.26 (±5.77)
BMI [kg/m^2^], mean (±SD)	30.07 (±5.48)	24.8 (±4.97)
Blood pressure [mmHg], mean (±SD)	129.03 (±22.23)/79.30 (±12.71)	119.67 (±8.59)/70.12 (±9.40)
Level of glycated hemoglobin (HbA1c), mean (±SD)	7.21 (±1.60)	-

t.2DM—type 2 diabetes; HbA1c—glycated hemoglobin; SD—standard deviation.

**Table 2:** Comparison of a group of patients with glycated hemoglobin below and above 7%.

	Patients with t.2DM	
Parameters	Patient group with HbA1c ≤ 7% n = 15	Patient group with HbA1c > 7% n = 15
Average level of glycated hemoglobin (HbA1c), mean (±SD)	6.11% (±0.39)	8.31% (±1.66)
Male/female ratio	6/9	6/9
Age (years), mean (±SD)	69.07 (±13.21)	70.20 (±14.55)
BMI [kg/m^2^], mean (±SD)	29.78 (±5.64)	30.67 (±5.20)
Duration of t.2DM (years), mean (±SD)	7.40 (±5.68)	15.6 (±14.08)
Blood pressure [mmHg], mean (±SD)	127.46 (±17.94)/80.27 (±8.10)	126.13 (±26.62)/75.87 (±15.69)
Heart rate: beats per minute [BPM], mean (±SD)	77.73 (±7.07)	78.53 (±15.90)

t.2DM—type 2 diabetes; HbA1c—glycated hemoglobin; F/M—female/male; SD—standard deviation.

**Table 3:** The strength of correlations between the level of glycated hemoglobin (HbA1c) in erythrocytes and the chosen tongue sectors’ temperature in the group of patients with diabetes. Statistically significant correlations (*p* < 0.05) are marked in bold.

Parameters of Thermal Imaging	Strength of Correlations HbA1c	*p*-Value
Tmean of tongue dorsum after 2 min	−0.1	
Tmean of tongue dorsum after 10 min	0.2	
ΔT of tongue dorsum	0.46	*p* < 0.05
Tmean of tongue apex after 2 min	0.05	
Tmean of tongue apex after 10 min	0.25	
ΔT of tongue apex	0.33	*p* < 0.05

## Data Availability

No data are unavailable due to privacy or ethical restrictions.
